# Violence experience among cis-gender women living with HIV in Atlanta, Georgia: impact on HIV-related health and their preferences for violence screening and support

**DOI:** 10.3389/fpubh.2025.1521493

**Published:** 2025-04-14

**Authors:** Sophia C. Garbarino, Katherine M. Anderson, Madelyn S. Carlson, Caroline W. Kokubun, Celeste K. Ellison, Selaem Hadera, Ameeta S. Kalokhe, Jessica M. Sales

**Affiliations:** ^1^Department of Behavioral, Social, and Health Education Sciences, Emory University Rollins School of Public Health, Atlanta, GA, United States; ^2^Department of Medicine, Emory University School of Medicine, Atlanta, GA, United States; ^3^Hubert Department of Global Health, Emory University Rollins School of Public Health, Atlanta, GA, United States

**Keywords:** intimate partner violence, non-partner violence, hate crimes, ACES, mental health, women living with HIV, viral suppression, retention in care

## Abstract

**Introduction:**

People living with HIV (PLWH) experience multiple forms of violence at higher rates than the general population; however, research on experiences of violence among cis-gender women living with HIV (CWLH) mainly focuses on intimate partner violence (IPV), with inconsistent documentation across the literature. To begin improving trauma-informed practices in HIV care, we examined experiences of IPV, non-partner violence (NPV), hate crimes, and adverse childhood experiences (ACEs) among CWLH. We then explored experiences and preferences regarding violence screening and support services among CWLH.

**Methods:**

As part of a larger study on violence experiences and screening among PLWH, 88 CWLH in Atlanta, Georgia, completed a cross-sectional survey on violence and mental health from February 2021 to December 2022 and provided consent for medical chart abstraction. A subgroup of 24 participants completed in-depth interviews on experiences and preferences related to violence screening. Univariate and bivariate analyses were used to assess violence prevalence and associations with mental health and chart-abstracted HIV outcomes. Thematic qualitative methods were employed for interview analysis.

**Results:**

Every participant (100%) experienced at least one form of violence in their lifetime, which included IPV among partnered CWLH (83.33%), NPV (96.51%), hate crimes (85.23%), and ACEs (80.68%). More than half of the participants (61.36%) met diagnostic criteria for at least one mental health condition. Multiple forms of violence had high co-occurrence with post-traumatic stress disorder, depression, viral suppression, and retainment in HIV care. Qualitative analysis revealed that most interview participants had discussed violence or trauma with a healthcare professional before, reporting a mix of positive and uncomfortable experiences. Participants offered diverse perspectives on improving the violence screening process, including recommendations on how, where, by whom, when, and how frequently screenings should occur.

**Conclusion:**

Multiple forms of violence are highly prevalent among CWLH, with several found to be associated with mental health and HIV outcomes. This highlights the necessity for a trauma-informed approach within HIV care settings. Healthcare professionals should consider the unique needs and preferences of CWLH when screening for violence and providing support services. Doing so may improve mental, physical, and overall well-being throughout the HIV care continuum.

## 1 Introduction

In the United States (US), over 1.1 million individuals live with HIV, with 23% identifying as women (1). Among individuals assigned female at birth, Black/African Americans (AAs) are disproportionately affected by HIV, accounting for 50% of new diagnoses in 2022 (1). In the US South, the epicenter of the HIV epidemic, the rate of HIV diagnosis per 100,000 is 20.7 among Black/AA females but only 2.7 among their White counterparts (2). Furthermore, Black/AAs make up 47% of all diagnoses despite comprising only 19% of the region’s population (1). These disparities have persisted over time and are attributed to institutional and social discrimination against the intersecting identities these individuals hold, including racism and gender-based discrimination (1). This includes discrimination relating to gender, race, and income across various levels, such as interpersonal, community, and systemic interactions (3).

As theorized in Singer’s Substance Abuse, Violence, and AIDS (SAVA) Syndemic theory (4), violence has been associated with poorer HIV outcomes. A significant portion of violence research concerning cis-gender women living with HIV (CWLH) centers on intimate partner violence (IPV), highlighting that they experience higher rates of IPV across various racial/ethnic, gender, and sexual identities (2, 3) compared to women in general (5). In a nationally representative sample of people living with HIV (PLWH), 26.3% reported having ever experienced intimate partner violence (IPV), with bisexual and heterosexual CWLH experiencing IPV at significantly higher rates than men at 51.5 and 35.3%, respectively (6). Several studies have examined the impact of IPV on HIV, in which IPV was consistently associated with poor HIV outcomes, including retention in care, medication adherence, and viral suppression (6). This may be especially pertinent among CWLH of color for two reasons. First, over half of Black (53.6%), American Indian/Alaska Native (57.7%), and multiracial (63.8%) women experience IPV in their lifetime (7). Second, racial/ethnic disparities in viral suppression outcomes may also exist; a national cohort study found that Black and Hispanic women were significantly less likely to achieve viral suppression each year from 2010 to 2015 (5).

However, this focus on IPV may lead to overlooking other forms of violence CWLH also experience, including non-partner violence (NPV; general, crime, and physical/sexual), hate crimes, and adverse childhood experiences (ACEs) (8–12). Unlike IPV, the relationships between these types of violence and HIV care outcomes among CWLH, including pathways mediated by substance use and mental health (13), remain unclear. While a cohort (9) and cross-sectional study (14) both found that NPV prevalence did not significantly differ between CWLH and women without HIV, approximately one third of each sample reported NPV. Currently, there are no national estimates for NPV prevalence among CWLH. Similarly, while emerging research has found low prevalence of hate crime experience among men who have sex with men (MSM) (15), little is known about hate crimes experienced by CWLH and what, if any, impact such experiences have on HIV outcomes. Carlson and colleagues conducted a multivariate regression analysis on the same dataset used in this study to examine the association between recent hate crime experiences and mental health and HIV outcomes among PLWH. Approximately half of the participants had experienced a hate crime in the previous year, and 93% had experienced hate-motivated violence in their lifetime. Among the survivors of lifetime hate violence (*N* = 262), nearly one-third were CWLH. The authors identified a significant association between recent hate violence and PTSD symptoms but no significant link with HIV outcomes (16).

Additionally, two studies involving PLWH, one focused on youth aged 17 to 24 in the US South (11) and the other in Washington state (10), revealed that while the prevalence of ACEs was high, there was no significant correlation between ACEs and HIV viral load. However, both study samples largely consisted of males and PLWH engaged in care during the study period; therefore, the relationship between ACEs and HIV outcomes may differ among CWLH and PLWH out of care or with reduced access to care.

Similarly, relatively little is known about the associations between non-intimate partner violence and mental health among PLWH. This gap is particularly concerning among CWLH, who experience depression (17, 18), anxiety (19), and post-traumatic stress disorder (PTSD) (20) at higher rates compared to men living with HIV or women living without HIV (21). A recent latent profile analysis found that Black CWLH experience greater severity of depression and PTSD when they face higher adversity (i.e., trauma, discrimination, and gender- or race-related microaggressions), while those with high resilience and low adversity had significantly lower depression, post-trauma cognition, and PTSD symptom scores compared to those with low resilience, suggesting resilience may buffer the relationship between trauma and HIV outcomes. However, the study did not identify any connections between mental health and specific trauma types, like partner versus non-partner trauma (22). Thus, while the relationship between mental health and HIV care has been extensively explored in public health (23), the violence, mental health, and HIV triad among CWLH is less understood.

Aligned with trauma-informed care (TIC), there is a growing call for implementing violence screening in HIV care settings, particularly IPV screening (24, 25). This includes recommendations from the White House (26), Health Resources and Services Administration (HRSA) HIV/AIDS Bureau (27), and Infectious Diseases Society of America (28). However, evidence on screening for other forms of violence remains limited, particularly on violence screening and support preferences among CWLH. Therefore, this study had two primary aims: (1) to assess the prevalence of violence experienced by CWLH across multiple forms (i.e., IPV, NPV, hate crimes, ACEs), including co-occurrence with mental health and HIV outcomes, to inform screening priorities, and (2) to better understand preferences for and recommendations related to violence screening among CWLH in Atlanta, Georgia.

## 2 Materials and methods

### 2.1 Study overview

The present study was part of a larger, multi-phase, mixed methods study of violence experiences and HIV care outcomes among PLWH. In Phase 1, we conducted a cross-sectional survey among PLWH (*N* = 285) in Atlanta, Georgia, to assess experiences of violence, mental health conditions, and HIV outcomes. In Phase 2, we conducted in-depth interviews with select survey participants (*N* = 69) to understand experiences and preferences surrounding violence screening practices. The current analysis includes only participants from each sample who identified as cisgender women (*N* = 88 surveys, *N* = 24 interviews).

### 2.2 Study setting

The US South region is the epicenter of the US HIV epidemic, accounting for 46% of the nation’s individuals living with diagnosed HIV (ages 13 and older) and 56% of HIV-related deaths (1). This region experiences the highest rate of poverty and lowest median household income, both of which contribute to HIV transmission (29). The state of Georgia has the second-highest rate of new diagnoses and third-highest prevalence rate at 23.0 and 558.7 per 100,000 people, respectively (1). Among individuals assigned female at birth aged 13 and older, Georgia has the second highest rate of new diagnoses at 10.6 per 100,000 (1). The Atlanta-Sandy Springs-Alpharetta metropolitan area, where this study took place, ranks third for new diagnosis rates among metropolitan areas (1).

The study was conducted in the four Ending the HIV Epidemic (EHE) (30) priority counties in Georgia: Fulton, Dekalb, Cobb, and Gwinnett, which experience high rates of income inequality, poverty, and racial segregation (31). Participants were recruited from 19 HIV service or research settings in these counties, including two hospital-affiliated Ryan White-funded clinics (RWCs) that served over 8,000 PLWH combined, eight independent RWCs, eight AIDS service organizations (ASOs), and one community-based clinical research site.

### 2.3 Eligibility and recruitment

In the larger study, eligible participants were living with HIV, at least 18 years old, had capacity to consent, and were fluent in either English or Spanish. For this analysis, participants also needed to identify as cisgender women. We employed purposive sampling to recruit people living with HIV, considering variations in race, ethnicity, and retention in HIV care [either retained in or out of care (OOC)]. Recruitment methods included in-person recruitment tables, flyers, and word-of-mouth. Recruitment efforts were supported by a Ryan White community advisory board (CAB) and ASO board, including troubleshooting support. In the hospital setting, participants were identified by reviewing inpatient social worker lists of admitted PLWH. Further details of recruitment procedures are available elsewhere (8).

Of the 88 survey participants from Phase 1, 73 indicated a willingness to be recontacted for an interview. From this pool of interested participants, individuals were purposively recruited for diversity of gender, race/ethnicity, violence exposure history, HIV viral suppression, and care engagement.

### 2.4 Data collection

Because our primary objective was to inform future violence screening practices, including previously underexplored forms of violence among CWLH (i.e., NPV, hate crimes, ACEs), we used mixed methods to capture violence prevalence and screening preferences among CWLH. Quantitative methods were used to assess prevalence, indicating which forms of violence were most common and thus should be covered in screenings, as well as co-occurrence with mental health and HIV care outcomes. Next, qualitative methods were used to elicit screening experiences and preferences (e.g., how to best ask about non-intimate partner forms of violence).

#### 2.4.1 Quantitative

Before taking part in Phase 1, all participants gave their written informed consent. This phase included an interviewer-administered survey, the collection of a 5-mL blood sample to measure HIV viral load, and completion of a Release of Information form and a Health Insurance Portability and Accountability Act (HIPAA) authorization form for reviewing medical records.

Surveys took approximately 60 min and were conducted from February 2021 to December 2022 using REDCap in a private one-on-one environment by trained study staff skilled in data collection and trauma-informed research methods (32–34). In line with trauma-informed methods, study staff prioritized participant privacy during the survey, including (1) informing participants that they could pause or exit the survey at any time; (2) pausing the survey and switching subjects to avoid disclosure of the study’s focus if privacy was disrupted; (3) establishing rapport prior to survey administration; and (4) providing a list of trauma, social, and community support services to all participants upon survey completion. Participants received $75 USD in cash for their involvement in the survey.

#### 2.4.2 Qualitative

A semi-structured interview guide was developed to facilitate discussions around preferences for and acceptability of violence screening practices. This included screening methods (e.g., in-person vs. paper-pencil), phrasing of screening items (e.g., direct vs. conversational), screening environment (e.g., by who, where), screening frequency (e.g., every visit, annually), and the timing of delivery (e.g., initial visit, subsequent visit). Between February 2022 and December 2022, interviews were facilitated by two study team members trained in qualitative and trauma-informed research methods. Interviews lasted approximately 30–60 min.

All participants provided written informed consent before the interviews and oral consent to be audio-recorded. The interviews were conducted in person at a centrally located research office, easily accessible by public transportation to minimize travel barriers for participants. To ensure that privacy and participant safety were maintained during the interview, all interviews were conducted in a private, one-on-one setting, and noise cancelation machines were used during the interviews. A resource guide with local trauma support and social services was available for all participants who requested information and to those who experienced distress during the interview. All participants received $50 USD in cash after the interview for their time and effort. Interviews were audio-recorded using a laptop, secure Zoom account and an audio tape recorder. All recordings were professionally transcribed verbatim by a third-party transcription service (Ubiqus). Transcripts were cleaned and de-identified by study team members.

### 2.5 Research ethics

All study procedures were approved by the university’s Institutional Review Board (IRB00117548). Further details on ethical procedures are available elsewhere (8).

### 2.6 Measures

#### 2.6.1 Participant background characteristics

Survey measures have been described in detail elsewhere (8). In brief, participants were asked to report demographic information, current alcohol use using the 10-item AUDIT (35, 36), past-year substance use using one item from the DAST (37) (“In the past 12 months, have you used any of the following drugs: solvents, tranquilizers, barbiturates, cocaine, crack, stimulants, hallucinogens, or narcotics?”), and cannabis use using a single item created by the study team (“In the past 12 months, have you used marijuana?”). Responses to substance use questions were dichotomized into *hazardous/harmful consumption or likely alcohol dependence* (yes/no; alcohol), and past-year use (yes/no; cannabis and non-cannabis drug use). Resilience was measured using the 25-item version of the Connor-Davidson Resilience Scale (CD-RISC), where higher scores indicate greater resilience on a 100-point scale (38). Finally, participants were asked whether they currently receive HIV care at a clinic, and if so, how they get there and how long it usually takes.

#### 2.6.2 Violence exposure

Violence survey measures have been described in detail elsewhere (8). In summary, the Childhood Trauma Questionnaire-Short Form (CTQ-SF), consisting of 28 items, assessed exposure to ACEs in two categories: neglect (including emotional and physical) and abuse (covering emotional, physical, and sexual). Each item offered a five-point response scale ranging from “Never True” (1) to “Very Often True” (5). A participant was considered to have experienced ACEs if they responded at least “Sometimes True” (2) to any question across the overall scale, sub-scale, or subtype sub-scale.

Lifetime and past-year IPV experience were measured using the Revised Conflict Tactics Scale (CTS2), a 39-item scale that captures frequency of *psychological* (eight items) and *physical* attacks (12 items), *sexual* coercion (seven items), physical *injury* (six items), and use of negotiation (six items) in intimate partnerships (39). Binary variables (any vs. none) were created for IPV overall and each subtype (psychological, physical, sexual, and injury).

Non-partner violence (NPV) experience and frequency was measured using the Trauma History Questionnaire (THQ), a 24-item questionnaire assessing traumatic lifetime events (40). We assessed three subtypes of NPV: crime (four items), general (13 items), and physical/sexual (seven items). Further details on the items used are available as Supplemental Material. Binary variables (any vs. none) were created for any NPV and each NPV subtype.

*Hate crime* experience was assessed using a 12-item, modified Anti-Gay Violence and Victimization scale, which incorporated other primary reasons for hate and discrimination (15, 41).The items reflected experiences that could have been influenced by prejudice, such as receiving verbal insults or being harassed by police (without assault). Participants were required to indicate their experiences as “never,” “at least once in my lifetime,” or “in the past year.” From this, binary variables for lifetime and past-year hate crime experience were established, coding responses of “at least once in my lifetime” or “in the past year” as yes for their respective variables.

#### 2.6.3 Mental health variables

*Post-traumatic stress disorder (PTSD)* was measured using the PTSD Checklist-Stressor Specific (PCL-S) for DSM-IV, a 17-item questionnaire assessing the presence of PTSD symptomatology (42). For each item, participants were asked to indicate how much they have been bothered by a symptom during the past month, with response options on a four-point scale from “Not at all” (1) to “Extremely” (5). Responses were summed (range: 17–85) and a binary variable was created where scores ≥30 indicated PTSD, aligning with the National Center for PTSD cutoff recommendation for civilian primary care (43).

*Depression* was measured using the Patient Health Questionnaire (PHQ-9), a nine-item instrument assessing the presence and severity of depression over the past two weeks (44). For each item, participants were asked to indicate the frequency of occurrence, with four response options from “Not at all” (0) to “Nearly every day” (3). Responses were summed (range: 0–27) and a binary variable was created where scores ≥10 indicated at least moderate depression (44).

*Anxiety* was measured using the GAD-7, a seven-item questionnaire assessing the presence and severity of generalized anxiety disorder (GAD) over the last two weeks (45). For each item, participants were asked to indicate the frequency of occurrence, with four response options from “Not at all” (0) to “Nearly every day” (3). Responses were summed (range: 0–21) and a binary variable was created anxiety where scores ≥8 indicated anxiety (46).

#### 2.6.4 HIV care outcomes

Participants were asked to sign a release of medical information for all clinics where they may have received HIV care. Our team made at least three attempts to obtain HIV primary care visit data from the clinic sites visited by participants. Trained study team members reviewed individual patient charts to extract visit data, performing quality assurance on approximately 22% of all chart abstractions. Viral suppression was determined using 200 copies/mL as the cutoff, aligning with the Centers for Disease Control and Prevention definition of viral suppression (47). Additionally, participants were asked to provide a 5-mL blood sample for HIV viral load at the time of survey data collection. Samples were processed by the institution’s clinical virology research laboratory.

Using extracted medical chart data and viral load at the time of visit, the following binary HIV care outcome variables were determined: *ever virally suppressed in the past 12* and *24 months*, *durably (sustained) virally suppressed in the past 12* and *24 months*, and *retained in HIV care over the past 24 months*. Viral suppression at enrollment was included within the past 12- and 24-month viral suppression variables. For *retention in HIV care over the past 24 months*, participants were coded as *yes* if they had attended at least one HIV care visit every six months in the past 24 months.

#### 2.6.5 Violence screening experiences and preferences

During in-depth interviews, participants were asked about experiences with and preferences for violence screening practices. Examples of screening experience questions included, “Have you ever talked to a healthcare professional about any personal experiences you have had with violence or trauma?” and if so, “Can you describe how you were asked about your experiences with violence at your clinic?” Screening preferences questions included, “How frequently do you think healthcare staff/providers should ask about violence-related experiences? Why?” and “Can you describe how you think providers/healthcare staff could make the violence screening process feel safer, supportive, and more comfortable?”

### 2.7 Data analysis

We conducted univariate and bivariate analyses using SAS 9.4 software. Median and interquartile ranges were reported for continuous variables (e.g., age). Frequency and percentages were reported for categorical variables (e.g., race); percentages represent prevalence out of those with available data. All bivariate analyses examined associations between violence exposure, mental health, and HIV care outcomes using Fisher’s exact test due to small sample and cell size (<5); significance was set at a threshold of *α* = 0.05. Missingness was limited (<5%) except travel time to HIV clinic (7.95% missing), ever virally suppressed in the past 12 (10.23%) and 24 (9.09%) months, durable viral suppression in the past 12 (10.23%) and 24 (19.32%) months, and retention in HIV care in the past 24 months (6.82%). This level of missingness is common in HIV viral load chart abstraction (48) and may be due to recruiting some participants out-of-care. IPV analyses were limited to individuals with a partner in the past 12 months (*N* = 48). We did not conduct multivariate analyses for variables due to small cell size.

For qualitative analysis, the study team iteratively developed a codebook consisting of deductive codes informed by the interview guide and inductive codes identified by reviewing a subset of transcripts and group discussions, in which code definitions were created and modified. The codebook was piloted on five transcripts, followed by coding checks, code definition refinement, and memo-making to reach team agreement on the codebook. Upon finalizing the codebook, a team of four, female study team members (including CWK and CKE) were assigned transcripts to code. Each transcript was assigned and independently coded by two study team members using MAXQDA 2022 software (49). To enhance the reliability and validity of the coding, the coding pairs rotated weekly. Intercoder agreement meetings were conducted among the coding pairs to identify discrepancies and reach a consensus for all transcripts. We then used thematic analysis techniques to determine patient preferences and acceptability surrounding violence screening implementation in RWC and ASO settings.

## 3 Results

### 3.1 Survey results

#### 3.1.1 Survey participant characteristics

As shown in Table 1, 88 CWLH completed the survey. Participants primarily identified as Black/AA (92.05%), non-Hispanic/Non-Latina (95.40%), and straight/heterosexual (90.91%). Participants had a mean age of 54.51 years old. Just over half (57.95%) reported having a high school education or less; 80.68% were unemployed; 55.68% were single/never married; 54.55% had a partner in the past 12 months; and 5.75 and 6.90% reported housing and food insecurity, respectively. In addition, 10.23% met the criteria for high-risk or likely dependent alcohol use, while 27.27 and 18.18% reported using cannabis or other drugs in the past 12 months, respectively. The median resilience score was 73 (possible range 0–100), and all but one participant (98.85%) had a clinic where they received HIV care.

**Table 1:** Characteristics of 88 cis-gender women living with HIV (CWLH), Atlanta, GA, 2021–2022.

Characteristic		*N* (%)
Age, median (IQR)		55 (13.50)
Race	Black / African American	81 (92.05)
	White	6 (6.82)
	Other	1 (1.14)
Ethnicity	Non-Hispanic/Non-Latina	83 (95.40)
Sexual identity	Bisexual	4 (4.55)
	Gay / queer / same gender loving / homosexual	2 (2.27)
	Straight / heterosexual	80 (90.91)
	Other	2 (2.27)
Education	College or additional education	7 (7.95)
	Some college, technical school, or vocational school	30 (34.09)
	High school or less	51 (57.95)
Employment	Unemployed	71 (80.68)
Household income*	Less than $10,000	4 (23.53)
	$10,000 – 19,999	4 (23.53)
	$20,000 – 39,999	4 (23.53)
	$40,000 – 59,999	3 (17.65)
	More than $60,000	2 (11.76)
Marital status	Single / never married	49 (55.68)
	Married / domestic partnership	11 (12.50)
	Widowed	4 (4.55)
	Divorced	24 (27.27)
Have you had a partner in the past 12 months?	Yes	48 (54.55)
Housing insecurity (spent at least one night in an overnight shelter and/or on the street without shelter, past six months)	Yes	5 (5.75)
In the last 30 days, was there any time when you did not get anything, or barely anything, to eat for two or more days?	Yes	6 (6.90)
Alcohol use (AUDIT, range 0–40)	High risk or likely dependent use (AUDIT ≥8)	9 (10.23)
Substance use. Past 12 months	Cannabis	24 (27.27)
	Solvents, tranquilizers, barbiturates, cocaine, crack, stimulants, hallucinogens, narcotics, or other drugs	16 (18.18)
Resilience score (CD-RISC, range 0–100)	Median (IQR)	73 (19.50)
Do you have a clinic where you receive HIV care?	Yes	86 (98.85)

AUDIT, alcohol use disorders identification test; CD-RISC, Connor-Davidson Resilience Scale.

*Among employed individuals, *N* = 17.

#### 3.1.2 Violence exposure and mental health

Most notably, every participant had experienced at least one form of violence in their lifetime (100%), with CWLH reporting high levels of lifetime NPV (96.51%), lifetime hate crimes (85.23%), and ACEs (80.68%). Among those with a partner in the past 12 months, most had experienced lifetime IPV (83.33%), and past-year IPV (82.98%). The most prevalent subtypes of violence were psychological IPV (83.33% lifetime, 82.98% past-year), general NPV (94.19%), and childhood abuse (84.09%). Over half (61.36%) met the threshold for at least one mental health condition; 54.55% had PTSD, 28.74% had depression, and 37.50% had anxiety. Frequencies and percentages for all violence exposures and subtypes are reported in Table 2.

**Table 2:** Violence exposure, mental health, and HIV care outcomes among 88 CWLH, Atlanta, GA, 2021–2022.

Violence exposure, mental health, and HIV care outcomes		*N* (%)
Any violence, lifetime		88 (100.00)
Intimate partner violence (IPV), lifetime*	Any*	40 (83.33)
	Psychological IPV*	40 (83.33)
	Assault IPV*	19 (39.58)
	Sexual IPV*	13 (27.08)
	Injury IPV*	10 (20.83)
Intimate partner violence (IPV), past 12 months*	Any*	39 (82.98)
	Psychological IPV*	39 (82.98)
	Assault IPV*	17 (35.42)
	Sexual IPV*	11 (23.40)
	Injury IPV*	8 (16.67)
Non-partner violence (NPV), lifetime	Any	83 (96.51)
	Crime	47 (53.41)
	General violence	81 (94.19)
	Physical or sexual violence	60 (68.18)
Hate crimes	Lifetime	75 (85.23)
	Past 12 months	29 (32.95)
Adverse childhood experiences (ACEs)	Any	71 (80.68)
	Any ACE, subtype: Abuse	74 (84.09)
	Abuse: Emotional	52 (59.77)
	Abuse: Physical	57 (65.52)
	Abuse: Sexual	59 (67.82)
	Any ACE, subtype: Neglect	52 (59.09)
	Neglect: Emotional	34 (40.00)
	Neglect: Physical	38 (43.18)
Mental health conditions	Any	54 (61.36)
	Post traumatic stress disorder (PTSD, PCL-S ≥ 30)	48 (54.55)
	Depression (PHQ-9 ≥ 10)	25 (28.74)
	Anxiety (GAD-7 ≥ 8)	33 (37.50)
HIV viral suppression	Ever virally suppressed, past 12 months	76 (96.20)
	Ever virally suppressed, past 24 months	79 (98.75)
	Durable viral suppression, past 12 months	68 (86.08)
	Durable viral suppression, past 24 months	59 (83.10)
Retained in HIV care, past 24 months		44 (53.66)

GAD-7, generalized anxiety disorder; PCL-S, PTSD checklist-stressor specific; PHQ-9, patient health questionnaire.

*Among individuals with a partner in the past 12 months, *N* = 48.

Certain forms of violence had high co-occurrence with mental health conditions. First, among those who met the threshold for PTSD (PCL-S ≥ 30), most reported any lifetime IPV (88.89%), any lifetime NPV (56.63%), lifetime hate crime (57.33%), and any ACEs (89.95%). Similarly, among those who met the threshold for depression (PHQ-9 ≥ 10), most reported lifetime IPV (87.5%) and any ACEs (92%). Lastly, among those who met the threshold for anxiety (GAD-7 ≥ 8), most reported lifetime IPV (90.48%) and any ACEs (87.88%). Co-occurrence of NPV and hate crime experience with mental health conditions was less prevalent.

All bivariate associations between violence exposure and mental health conditions are reported in Table 3. Bolded font indicates *p* < 0.05. However, statistical significance should be regarded with caution because our sample was small, and we were unable to conduct multivariate analysis. Overall, multiple forms of violence were independently associated with having PTSD, including all lifetime NPV subtypes, past-year hate crime, and any childhood abuse. Lifetime and past-year assault IPV and childhood physical abuse were associated with depression. Finally, only childhood physical abuse was associated with anxiety.

**Table 3:** Violence exposure and mental health conditions among 88 CWLH, Atlanta, GA, 2021–2022.

	Mental health condition, *N* (%)					
Violence exposure	Post-traumatic stress disorder (PTSD)		Depression		Anxiety	
	Yes	No	Yes	No	Yes	No
Any intimate partner violence (IPV), lifetime*	24 (88.89)	16 (76.19)	14 (87.50)	25 (80.65)	19 (90.48)	21 (77.78)
Psychological IPV*	24 (88.89)	16 (76.19)	14 (87.50)	25 (80.65)	19 (90.48)	21 (77.78)
Assault IPV*	13 (48.15)	6 (28.57)	**10 (62.50)**	**9 (29.03)**	10 (47.62)	9 (33.33)
Sexual IPV*	8 (29.63)	5 (23.81)	5 (31.25)	7 (22.58)	6 (28.57)	7 (25.93)
Injury IPV*	7 (25.93)	3 (14.29)	4 (25.00)	6 (19.35)	5 (23.81)	5 (18.52)
Any intimate partner violence (IPV), past 12 months*	23 (58.97)	16 (41.03)	13 (34.21)	25 (65.79)	19 (48.72)	20 (51.28)
Psychological IPV*	23 (58.97)	16 (41.03)	13 (34.21)	25 (65.79)	19 (48.72)	20 (51.28)
Assault IPV*	12 (70.59)	5 (29.41)	**9 (52.94)**	**8 (47.06)**	10 (58.82)	7 (41.18)
Sexual IPV*	7 (63.64)	4 (36.36)	4 (40.00)	6 (60.00)	6 (54.55)	5 (45.45)
Injury IPV*	5 (62.50)	3 (37.50)	3 (37.50)	5 (62.50)	5 (62.50)	3 (37.50)
Any non-partner violence (NPV), lifetime	47 (56.63)	36 (43.37)	25 (30.49)	57 (69.51)	32 (38.55)	51 (61.45)
Crime	**30 (63.83)**	**17 (36.17)**	15 (32.61)	31 (67.39)	20 (42.55)	27 (57.45)
General violence	**47 (58.02)**	**34 (41.98)**	25 (31.25)	55 (68.75)	32 (39.51)	49 (60.49)
Physical or sexual violence	**38 (63.33)**	**22 (36.67)**	18 (30.51)	41 (69.49)	25 (41.67)	35 (58.33)
Hate crime						
Lifetime	43 (57.33)	32 (42.67)	21 (28.00)	54 (72.00)	28 (37.33)	47 (62.67)
Past 12 months	**22 (75.86)**	**7 (24.14)**	10 (34.48)	19 (65.52)	13 (44.83)	16 (55.17)
Any adverse childhood experience (ACE)	43 (89.58)	31 (77.50)	23 (92.00)	50 (80.65)	29 (87.88)	45 (81.82)
Any ACE, subtype: abuse	**43 (89.58)**	**28 (70.00)**	23 (92.00)	47 (75.81)	29 (87.88)	42 (76.36)
Abuse: emotional	**34 (72.34)**	**18 (45.00)**	17 (70.83)	35 (56.45)	19 (59.38)	33 (60.00)
Abuse: physical	**38 (80.85)**	**19 (47.50)**	**20 (83.33)**	**36 (58.06)**	**26 (81.25)**	**31 (56.36)**
Abuse: sexual	35 (74.47)	24 (60.00)	18 (75.00)	41 (66.13)	24 (72.73)	35 (64.81)
Any ACE, subtype: neglect	31 (64.58)	21 (52.50)	16 (64.00)	36 (58.06)	16 (48.48)	36 (65.45)
Neglect: emotional	21 (46.67)	13 (32.50)	11 (47.83)	23 (37.70)	11 (34.38)	23 (43.40)
Neglect: physical	21 (43.75)	17 (42.50)	9 (36.00)	29 (46.77)	11 (33.33)	27 (49.09

*Among individuals with a partner in the past 12 months, *N* = 48. Bolded values were statistically significant (*p* < 0.05).

#### 3.1.3 HIV care outcomes

As presented in Table 2, most participants had been virally suppressed at some point in the past 12 months (98.75%) and 24 months (96.20%). Durable viral suppression was slightly lower at 86.08 and 83.10% for the past 12 and 24 months, respectively. About half (53.66%) of participants were retained in HIV care over the past 24 months.

Co-occurrence of violence and lack of viral suppression was low; however, this was due to extremely low variability in viral suppression outcomes rather than lack of violence exposure. Our sample primarily comprised CWLH who had sustained viral suppression over the past 12 and 24 months (86.08 and 83.10%, respectively) and had a clinic where they received HIV care (98.85%). The fifth HIV care outcome, retention in care, had more variability. Among those who were not retained in care over the past 24 months, most reported any lifetime IPV (86.96%), any lifetime NPV (94.59%), lifetime hate crime (89.47%), and any ACEs (86.84%).

All bivariate associations between violence exposures and HIV care outcomes are shown in Table 4. Bolded font indicates *p* < 0.05. However, statistical significance should be regarded with caution because our sample was small, and we were unable to conduct multivariate analysis. No forms of violence were associated with ever having been virally suppressed in the past 12 or 24 months. Only lifetime physical/sexual NPV was associated with durable viral suppression in the past 12 months, while past-year hate crime was associated with durable viral suppression in the past 24 months. Finally, lifetime crime and physical/sexual NPV and any childhood neglect were associated with retention in HIV care over the past 24 months.

**Table 4:** Violence exposure and HIV care outcomes among 88 CWLH, Atlanta, GA, 2021–2022.

	HIV care outcome, *N* (%)									
Violence exposure	Ever virally suppressed, past 12 months		Ever virally suppressed, past 24 months		Durable viral suppression, past 12 months		Durable viral suppression, past 24 months		Retained in HIV care, past 24 months	
	Yes	No	Yes	No	Yes	No	Yes	No	Yes	No
Any intimate partner violence (IPV), lifetime*	34 (80.95)	2 (100.00)	36 (81.82)	1 (100.00)	30 (81.08)	6 (85.71)	24 (80.00)	8 (88.89)	18 (78.26)	20 (86.96)
Psychological IPV*	34 (80.95)	2 (100.00)	36 (81.82)	1 (100.00)	30 (81.08)	6 (85.71)	24 (80.00)	8 (88.89)	18 (78.26)	20 (86.96)
Assault IPV*	14 (33.33)	1 (50.00)	15 (34.09)	1 (100.00)	13 (35.14)	2 (28.57)	9 (30.00)	4 (44.44)	11 (47.83)	6 (26.09)
Sexual IPV*	12 (28.57)	1 (50.00)	13 (29.55)	0 (0.00)	11 (29.73)	2 (28.57)	8 (26.67)	3 (33.33)	8 (34.78)	5 (21.74)
Injury IPV*	7 (16.67)	0 (0.00)	7 (15.91)	0 (0.00)	1 (14.29)	6 (16.22)	5 (16.67)	0 (0.00)	5 (21.74)	3 (13.04)
Any intimate partner violence (IPV), past 12 months*	33 (80.49)	2 (100.00)	35 (81.40)	1 (100.00)	29 (80.56)	6 (85.71)	23 (79.31)	8 (88.89)	20 (86.96)	17 (77.27)
Psychological IPV*	33 (80.49)	2 (100.00)	35 (81.40)	1 (100.00)	29 (80.56)	6 (85.71)	23 (79.31)	8 (88.89)	20 (86.96)	17 (77.27)
Assault IPV*	12 (28.57)	1 (50.00)	13 (29.55)	1 (100.00)	11 (29.73)	2 (28.57)	7 (23.33)	4 (44.44)	6 (26.09)	9 (39.13)
Sexual IPV*	10 (24.39)	1 (50.00)	11 (25.58)	0 (0.00)	9 (25.00)	2 (28.57)	6 (20.69)	3 (33.33)	5 (22.73)	6 (26.09)
Injury IPV*	5 (11.90)	0 (0.00)	5 (11.90)	0 (0.00)	4 (10.81)	1 (14.29)	4 (13.33)	0 (0.00)	3 (13.04)	3 (13.04)
Any non-partner violence (NPV), lifetime	71 (95.95)	3 (100.00)	74 (96.10)	1 (100.00)	64 (95.52)	10 (100.00)	56 (96.55)	11 (100.00)	42 (97.67)	35 (94.59)
Crime	41 (53.95)	2 (66.67)	43 (54.53)	0 (0.00)	37 (54.41)	6 (54.55)	30 (50.85)	5 (41.67)	**19 (43.18)**	**25 (65.79)**
General violence	69 (93.24)	3 (100.00)	72 (93.51)	1 (100.00)	62 (92.54)	10 (100.00)	55 (94.83)	11 (100.00)	41 (95.35)	34 (91.89)
Physical or sexual violence	50 (65.79)	2 (66.67)	52 (65.82)	1 (100.00)	**42 (61.76)**	**10 (90.91)**	36 (61.02)	10 (83.33)	**26 (59.09)**	**29 (76.32)**
Hate crime										
Lifetime	63 (82.89)	3 (100.00)	66 (83.54)	1 (100.00)	55 (80.88)	11 (100.00)	47 (79.66)	12 (100.00)	35 (79.55)	34 (89.47)
Past 12 months	23 (30.26)	2 (66.67)	24 (30.38)	1 (100.00)	19 (27.94)	6 (54.55)	**16 (27.12)**	**7 (58.33)**	14 (31.82)	13 (34.21)
Any adverse childhood experience (ACE)	64 (84.21)	1 (33.33)	66 (83.54)	1 (100.00)	57 (83.82)	8 (72.73)	48 (81.36)	10 (83.33)	35 (79.55)	33 (86.84)
Any ACE, subtype: abuse	61 (80.26)	1 (33.33)	63 (79.75)	1 (100.00)	54 (79.41)	8 (72.373)	45 (76.27)	10 (83.33)	34 (77.27)	31 (81.58)
Abuse: emotional	45 (60.00)	0 (0.00)	46 (58.97)	0 (0.00)	40 (58.82)	5 (50.00)	34 (57.63)	5 (41.67)	23 (52.57)	24 (64.86)
Abuse: physical	49 (64.47)	0 (0.00)	51 (64.56)	0	43 (63.24)	6 (60.00)	35 (59.32)	7 (63.64)	27 (62.79)	25 (65.79)
Abuse: sexual	50 (66.67)	1 (33.33)	52 (66.67)	1 (100.00)	43 (64.18)	8 (72.73)	37 (62.71)	8 (66.67)	26 (59.09)	27 (72.97)
Any ACE, subtype: neglect	47 (61.84)	0 (0.00)	46 (60.76)	0 (0.00)	40 (58.82)	7 (63.64)	33 (55.93)	7 (58.33)	**22 (50.00)**	**26 (68.42)**
Neglect: emotional	31 (41.33)	0 (0.00)	31 (40.26)	0 (0.00)	26 (38.81)	5 (45.45)	21 (36.21)	5 (45.45)	14 (31.82)	17 (47.22)
Neglect: physical	34 (44.74)	0 (0.00)	35 (44.30)	0 (0.00)	29 (42.65)	5 (45.45)	25 (42.37)	5 (41.67)	18 (40.91)	17 (44.74)

* Among individuals with a partner in the past 12 months, *N* = 48. Bolded values were statistically significant (*p* < 0.05).

### 3.2 In-depth interview results

#### 3.2.1 In-depth interview participant characteristics

As presented in Table 5, 24 CWLH participated in in-depth interviews. The majority identified as Black/African American (*N* = 21), all were Non-Hispanic/Latina (*N* = 24), and all participants with available data had a history of violence and/or trauma (*N* = 23, missing for one participant). Nearly every participant was virally suppressed at the time of survey data collection (*N* = 22).

**Table 5:** Characteristics of 24 CWLH who completed in-depth interviews, Atlanta, GA, 2022.

Characteristic		N (%)
Age, median (IQR)		57 (13.00)
Race	Black / African American	21 (87.50)
	White	3 (12.50)
Non-Hispanic / Non-Latina	Yes	24 (100.00)
History of violence and/or trauma	Yes	23 (100.00)
Virally suppressed at time of survey data collection	Yes	22 (91.67)
Have you ever talked to a healthcare professional about any personal experiences you have had with violence or trauma?	Any	17 (70.83)
	Formal screening	4 (16.67)
In your experience, have you ever felt unsafe, unsupported, or uncomfortable during the violence screening process?	Yes	6 (23.08)

#### 3.2.2 Violence screening experiences

Approximately 71% (*n* = 17) of participants reported discussing their experiences with violence or trauma with healthcare professionals. Participants had spoken to various health workers about violence/trauma experiences, including clinical providers, therapists, counselors, case managers, and social workers. When asked what prompted the conversation about violence/trauma, only four reported being formally screened:

“I opened up because they have an assessment tool where you have to list stuff like that [violence experiences]. I listed that down and I briefly touched on it with [my provider].” – Participant 248, Black/AA.

A few had self-volunteered to disclose experiences with violence because of physical harm, poor mental health, or a sign in the doctor’s office:

“One night coming in from work, I was riding the bus, and it was a 3 to 11 shift, and a guy stopped me… He grabbed me and he had a knife, um he stabbed me several times… I did talk to my health provider about that…I wanted her to look at those wounds, to make sure, you know, it wasn’t getting infected or something.” – Participant 284, Black/AA.

“I needed help. And I was mentally going through it at that time. So I talked to my doctor at that time about it. Told about what was going on, what trauma. Because I’ve been raped. I’ve been robbed. And those were some very scary times with me.” – Participant 299, Black/AA.

“Their [doctor’s office]” signage is like, ‘If you are experiencing this [domestic violence], call this number, if you do not want to speak to somebody.’” – Participant 012, White.

Others disclosed violence experiences in response to questions that were not specific to violence but perhaps related (e.g., marriage, PTSD, substance use):

“I told her that I was married and [the counselor] said ‘what kind of marriage did you have?’ And I was like, ‘he was very abusive, girl’ and it all went on to talk about it…” – Participant 150, Black/AA.

Overall, most participants viewed these conversations about violence positively. They felt comfortable that the conversation was not rushed or forced and that it was “helping me. Getting it out” (Participant 210, Black/AA). A peaceful environment and a good relationship with their provider were also helpful in cultivating a positive experience.

“It was like - it was calm and cool. She was like, would I like to get further on into the conversation and I said, ‘Yes.’ She said I did not have to discuss it now, but whenever I was ready to go further in it, it was all right.” – Participant 309, Black/AA.

By contrast, six participants (25%) reported feeling unsafe, unsupported, or uncomfortable during the violence screening process. These women characterized the violence screening process—whether formal or informal—as re-traumatizing, triggering, and/or unsafe, citing issues like open exam room doors or pressure from healthcare professionals to disclose information. They reported experiences of anger directed at them and not being taken seriously regarding their situations. One participant described a particularly harmful experience with a provider that occurred while her abuser was present in the waiting room:

“So and then this doctor was laughing about it [violence screening process]. I do not think that’s funny. That’s something very serious. Do not do that to a person. And then they, this doctor was talking real loud and I kept telling him, ‘Can you lower your voice ‘cause the person is out here?’ … I said, ‘I cannot see you no more… You might be a good doctor but I do not feel safe with you because you are about to blow everything out the ocean.’… I was scared he was going to get me killed because you do not know if that abuser is by that door…” – Participant 017, White.

#### 3.2.3 Violence screening preferences

##### 3.2.3.1 How and where should violence screening be conducted?

When asked how they would prefer to be screened, most participants preferred in-person, one-on-one discussions with a healthcare worker because face-to-face conversations were more comfortable, informal, and less frustrating than questionnaires. Several participants also noted it was easier to read body language and express emotions for both patients and providers, as well as allow providers to recognize physical signs of violence:

“Well, I always think it should be done in person, because you can – I’m pretty sure that people who are experienced in talking about violence in relationships can read body language, can read facial expressions, because sometimes a person might be afraid to tell you what’s going on, and then you can maybe probe or give them time and come back to it.” – Participant 001, Black/AA.

While a few preferred a one-on-one phone call or virtual meeting (e.g., Zoom), some were concerned that these methods are not always a safe environment to disclose violence:

“Over the phone is okay, but it’s whether or not the victim would be safe … ‘Cause [if an abuser hears] then they are gonna [say], ‘Oh, you told somebody about us?’ And then we’ll get beat up on more… That’s when they get more abusive, and that’s how, y’know, a lot of times women end up dead.” – Participant 012, White.

Others preferred a screener form via computer, email, or paper over conversation. Those who preferred a form explained that it would be more private, could be done at the patient’s pace, and would not force patients to say things aloud. Still, several participants suggested having multiple options available “each person would be different” (Participant 012, White).

##### 3.2.3.2 Who should conduct violence screening?

Participants expressed varied preferences regarding who should conduct violence screenings. Though most preferred a clinical or mental health provider, a wide range of other healthcare roles, including nurses, medical assistants, social workers, and case managers, were acceptable. Two participants also noted that a peer could conduct the screening if they had lived experience with violence.

Many participants emphasized the importance of certain traits over the screener’s specific role. In addition to lived experience, participants preferred a person who had mental health or other relevant training, used “discretion,” and was “familiar,” “empathetic,” “compassionate,” “nonjudgmental,” “understanding,” “respectful,” “genuine,” “willing to listen,” and “knowledgeable of that certain area which is HIV and HIV violence.” When asked about preferred demographics, including age, gender, and race of the person conducting the screening, participants again had diverse preferences. Ten participants strongly preferred women and/or someone of similar age, while eight said age, race, and gender did not matter:

“A woman [should conduct the screening] for a woman [patient] because most women do not want to talk to a man… I do not want to talk to a man about me getting beat up. Me personally, I would not. I do not think a lot of women would… Not too young. I do not like them fresh out of college when they want to sit down with a 20-year-old and have a conversation.” – Participant 218, Black/AA.

“As long as there’s comprehension with communication. They’re able to like I was saying get that connection with you regardless of age, race, color, that. Yes. I think I would not matter who asked. ‘Cause in all it’s supposed to be from the heart. Supposed to be genuine.” – Participant 090, Black/AA.

Only one participant explicitly said she preferred a Black woman like herself, and only one preferred a male to conduct the violence screening. Generally, characteristics such as compassion were valued more than demographic concordance.

##### 3.2.3.3 When and how often should violence screening be conducted?

When asked when violence screening should be conducted, participants expressed a range of preferences. Eleven participants suggested screening upon diagnosis, intake, and/or check-in to facilitate early referral to resources if needed. One participant discussed how violence may have caused the patient to acquire HIV, making early disclosure critical to engaging in HIV care. Others did not specify exact timing for the screening process. Instead, they expressed a preference for screenings to take place “before seeing my provider,” “when I feel ready,” or after a trusting relationship is formed between the patient and the healthcare professional.

When asked how often violence screening should be conducted, participants were split between every visit, every three to four months, once or twice per year, and on an “as needed” basis (e.g., when a provider recognizes signs/symptoms of violence, patient falls out of care, patient begins a new relationship). Those who preferred every visit or every three to four months justified their preference with frequently changing safety, relationship, and mental health status:

“I’m not going to tell you every appointment but every couple months, because you never know what a person is going through, especially individuals that’s not stable in housing and mentally… Like me, I wish someone would talk to me more about in the beginning about violence and HIV. That would have been a better outlook and a better relief for me if someone would have talked to me more often to find out who I am and how I got to this point and would have talked to me maybe every three months about like I said in the past or the present of violence dealing with HIV.” – Participant 028, Black/AA.

In contrast, those who preferred a once per year, twice per year, or on an as-needed basis expressed concern that screening too frequently could lead to frustration and/or re-traumatization:

“It still hurts… I do not know if you should address it every time because sometimes like I get with myself and I put it in the back of mind the more I have to deal with it the more it hurts. – Participant 278, White.

Several participants emphasized the importance of adapting screening frequency to patient needs and preferences:

“It should be up to the individual if they want to talk about it or how often they want to talk about it.” – Participant 150, Black/AA.

##### 3.2.3.4 What questions should be asked during violence screening?

When asked what types of questions should be asked during violence screening, many participants felt that beginning with a checklist or questionnaire would be acceptable. However, they also emphasized the importance of following the natural flow of conversation. Some suggested starting with general questions to allow for more specific follow ups:

“Just slide it in. Have a conversation about regular life and what’s going on in their life. If they are joking and smiling, you do not have any concerns. But if they are like well I did this and went to bed and I could not sleep last night, then you can start to following up with that.” – Participant 218, Black/AA.

Once rapport has been established, most participants suggested asking about multiple types of violence, including childhood/past, present, physical, sexual, verbal/emotional, partner/domestic, and non-partner, as well as the direction of violence (i.e., patient being the victim, perpetrator, or both). Participants were split between question wording preferences. Some desired more “straightforward” wording using potentially triggering language, such as:

“Have you ever been physically assaulted?” – Participant 248, Black/AA.

“Have you been raped? Have you been molested? Have you been in domestic violence? Are you being abused?” – Participant 299, Black/AA.

“Have you ever been abused in your childhood/adulthood?” Participant 017, white.

“Has anyone harmed you? Have you harmed anyone?” – Participant 306, Black/AA.

Others preferred questions to be worded without such language, either to avoid a “trigger” or to empower patients to discuss their experiences, such as:

“Are you safe? Is there anything in your home that is going on that you want me to know about?” – Participant 287, Black/AA.

“I was asked a few questions that really made me think, but they were very good. ‘Is there anybody touching you in a manner that you do not wanna be touched?’ Or, ‘Is there anybody that violates your personal space without your permission?’ That gave me the power. Instead of saying, ‘Somebody else was forcing you,’ for me to say, ‘Yes, this person touches me when I do not wanna be touched.’ And, ‘Yes, I feel this type of way because of the way this person treats me.’ It gave me a voice in my situation. ‘Cause sometimes the victims, we do not have a voice.” – Participant 012, White.

One participant further discussed her preference for brevity, as it allowed her space to disclose as much as she was comfortable with:

“I ain’t all that good with all them questions. Just ask me one question and let me answer, going all over. ‘Cause then I get frustrated. I’m that kind of person that if I get too many questions thrown at me I get frustrated because then I’ll be like oh I’m on a witness stand or something.” – Participant 150, Black/AA.

Some participants suggested that the person conducting the screening should provide support or ask about resources available to the patient. For example:

“And what do you want to do in order to make the situation better? And what can I do? How can I help in order to make the situation better for you?” – Participant 299, Black/AA.

“Do you have any support services? Seeking psychiatric treatment? Do you have a safe place to go if you need to? Do you have anybody that you can speak to if you are in danger? Are there children involved in the domestic violence situation? Would you be able to have transportation to get away from the situation if you needed to?” – Participant 279, Black/AA.

#### 3.2.4 How can healthcare professionals improve the violence screening process?

“I have to feel soothed. I have to feel safe.” – Participant 248, Black/AA.

When asked how providers and healthcare staff could make the violence screening process feel safer, supportive, and more comfortable, participants emphasized the importance of creating a safe space. Physically safe spaces were characterized as being “comfortable” (e.g., comfortable chairs, “quiet” room), “relaxed,” “calm,” “confidential,” “private,” and secure (e.g., having “security” personnel on site):

“… they changed the whole building… [before,] It brought out the sadness of why you are there. So I’m glad like they brightened up the rooms and the facility in itself because when you walked in there it just made you just- ‘ugh, I’m here.’ Now it’s more inviting. I would like to be somewhere where its more inviting and more comfortable for myself.” – Participant 028, Black/AA.

Creating an emotionally safe space was especially salient. All 24 participants discussed the need for healthcare professionals to be emotionally supportive. In addition to being “understanding,” “caring,” compassionate, empathetic, “considerate,” and attentive to patients’ needs, participants emphasized that healthcare professionals, particularly clinical and mental health providers, should not rush the conversation, force patients to discuss violence if they do not feel ready, or generalize across experiences of violence:

“They just put us all in a group and assume that we have all been in the same domestic violence situations, and a lot of times because of that, we tend to shy away and back off and not try to get help because they are generalizing it and putting us all in one category.” – Participant 279, Black/AA.

Instead, participants preferred that healthcare professionals “meet me where I’m at” (Participant 150, Black/AA) and practice active listening with “open ears” (Participant 152, Black/AA). Many also discussed having additional resources available on standby or facilitating referrals (e.g., support group, social worker) when desired:

“Have someone you know, ready and able to speak with you right away. And that’s it, you know. Or pull them to the side. Have someone ready and available to speak with you that day [i.e., mental healthcare professional].” – Participant 211, Black/AA.

“It’s like, ‘Here’s an apple pie if you wanna eat apple pie. But if you do not wanna eat apple pie, it’s okay. We’re still gonna take care of you…’ And it’s just, y’know, if you cannot force a victim to take care, but if we know it’s there, it makes it easier to reach out for the care.” – Participant 012, White.

One participant cautioned against screening for violence without proper referral channels or resources in place:

“I would not want anybody coming to me and saying oh we learned about this domestic situation that you are in. But then you are not going to do anything to help. If you do not have any help do not come and ask me. Let me help you get out of that situation. Immediately how can we help? If you are not doing that, if you are not in a position to do it, do not ask me. If you cannot help me so be it.” – Participant 218, Black/AA.

## 4 Discussion

### 4.1 Violence prevalence

Though high rates of violence and trauma among CWLH are well documented (20), most of the available research has centered around IPV (50, 51) and ACEs (10, 52). Few studies examine other types of violence, such as NPV (14) and hate crimes (8). Using standardized measures, our study comprehensively reports these forms of violence experienced by CWLH across a single sample. Identifying co-occurring experiences of violence broadens our knowledge of violence prevalence among CWLH, allowing for triangularization among the growing body of literature on rates of violence for CWLH. This is especially important among Black/AA CWLH, who lie at the heart of the US HIV epidemic in the US South (2).

Nearly all forms of violence measured (including subtypes) were highly prevalent across our sample. These findings are similar, if not higher than those reported in other studies of CWLH. Starting with IPV, on which most violence literature among CWLH focuses, nearly all our sample’s partnered individuals reported experiencing IPV in their lifetime (83.33%) and in the past 12 months (82.98%). These findings are substantially higher than those reported in other studies. For example, a multisite US study of 564 CWLH of color found that 27.3% reported lifetime IPV (53). Additionally, one recent meta-analysis found the lifetime IPV prevalence to be 42% (any), 29% (physical), and 20% (sexual) among CWLH globally (50). While the included CWLH studies varied in sample size and instruments for measuring IPV, those employing different versions of the CTS reported comparably lower IPV prevalence rates, ranging from 13.7 to 71% (49–58). The highest prevalence of 71% came from the sole US-based study (54). Such high prevalence may be due to the study’s sample size (*N* = 64), which was smaller than other included studies among CWLH using the CTS (50); additionally, the sample comprised mostly Black CWLH (51.6%) whose partners were not living with HIV, creating an unequal, gender- and diagnosis-based power dynamic (54).

Prevalence of NPV and hate crime experiences have been notably less explored among CWLH. However, our sample had substantially higher rates of lifetime NPV than the few others found in the literature. Nearly all (96.51%) reported any lifetime NPV. In comparison, a study of 249 Canadian CWLH found that 52 and 41% had experienced NPV (physical, sexual, or verbal) perpetrated by a stranger or acquaintance, respectively (55). Furthermore, 68.18% of our sample reported physical/sexual NPV, compared to a 2003 study of 310 CWLH in which under a third reported physical (29%) and sexual (17%) NPV in adulthood (14). Among a larger US study of 2,098 CWLH, 27% reported non-partner sexual abuse occurring at age 13 or later (9). Variation in prevalence across these studies may be due to measurement variability, as we used measured NPV using the 24-item THQ (see Supplemental Material) while others used unstandardized measures (14, 55) or only two items from the Revised CTS (9). Additionally, to our knowledge, ours is the first to capture hate crimes experience among CWLH, aside from our recent publication of violence experiences among PLWH in our larger study sample, which included PLWH with other gender identities (8).

Finally, 80.68% of our sample reported at least one ACE, with over half indicating childhood emotional (59.77%), physical (65.52%), and sexual (67.82%) abuse. These rates are substantially higher than estimates for the general US female population: 33.93% (emotional), 17.53% (physical), and 16.33% (sexual) (56). ACE prevalence among CWLH in other studies varies. For example, in a study of 95 South African CWLH, all (100%) reported any childhood trauma (52). Our findings are also slightly higher but in line with two US-based studies of PLWH who use substances. In one study, over half of female participants reported childhood sexual (51%) and physical (64%) abuse (57), and in another, 45.8% of CWLH reported childhood sexual abuse (58). High ACEs prevalence across these studies, including ours, is unsurprising given that unemployment and low income, to which ACEs have been linked within the general population (59, 60), were also prevalent (52, 57, 58).

Overall, the prevalence of IPV, NPV, and ACEs in our sample was higher and/or comparable to those found in the literature, suggesting these forms of violence are commonly experienced among CWLH. Additionally, all forms of non-intimate partner violence (NPV, hate crimes, and ACEs) were highly prevalent but are often not prioritized in screening practices in healthcare settings. For example, HRSA’s 2013 *Guide to Clinical Care of Women with HIV* emphasizes the importance of screening for domestic violence but not other forms of violence (61). HRSA’s guide also identifies depression and other aspects of mental health as key components of health and well-being among CWLH (61); however, these concerns cannot be fully understood nor addressed without recognizing the contexts in which they occur (i.e., violence).

### 4.2 Violence, mental health, and HIV care outcomes

Numerous studies have identified connections among violence and HIV care outcomes, especially through the lens of the SAVA Syndemic theory (12–14, 62). Additionally, while mental health is associated with HIV care outcomes (63–66), less understood are links between violence and mental health among PLWH This study explored the bivariate relationships among various types of violence, mental health, and HIV care outcomes, though quantitative results should be interpreted with caution due to lack of multivariate analysis and small sample size.

Our study contributes to an emerging body of mixed evidence on violence and mental health among CWLH. Most notably, multiple forms of violence co-occurred with PTSD, depression, anxiety, and lack of 24-month retention in HIV care. Most bivariate relationships were not significant (*p* > 0.05). However, NPV subtypes, past-year hate crime, and emotional and physical childhood abuse were each associated with PTSD. In comparison, a study of Black CWLH found higher PTSD symptom prevalence among those with more experiences of trauma (22), which is somewhat consistent with our findings. However, a study of South African CWLH of similar sample size found no association between IPV and PTSD (67). Future research should synthesize findings to explore such variation across studies. Additionally, assault IPV and childhood physical abuse were both associated with depression, while only childhood physical abuse was associated with anxiety. Though research linking childhood trauma and anxiety among CWLH is limited, a recent study of PLWH found that experiencing at least one ACE was associated with greater odds of anxiety (62). In turn, anxiety may mediate the relationship between violence and HIV care outcomes among CWLH (63). We did not explore this within our cross-sectional sample, but future studies should examine this potential mechanism.

IPV was not significantly associated with any HIV care outcomes. However, a robust body of evidence has established that IPV exposure increases the odds of poor HIV outcomes (51, 64). Less understood are the mechanisms through which IPV impacts HIV outcomes. Antiretroviral therapy (ART) adherence has been identified as a potential pathway; for example, a scoping review found that CWLH reported ART nonadherence due to IPV poor mental health caused by IPV and/or abusive partners preventing them from taking ARTs (65). While we did not measure ART in this study, our qualitative findings suggest that CWLH with abusive partners necessarily prioritized personal safety over HIV care. Further research should explore other potential drivers of low HIV care engagement and access among CWLH, including prioritizing the safety of one’s child, housing insecurity, food insecurity, and poor social support.

We also found that physical/sexual NPV was associated with durable 12-month viral suppression and 24-month retention in HIV care. Also, past-year hate crime experience was associated with durable 24-month viral suppression. All other NPV and hate crime relationships were not significant. To our knowledge, our study is among the first to report any relationships between NPV, hate crimes, and HIV care outcomes among CWLH. However, our analyses were limited by low variability in viral suppression outcomes. Lastly, experience of any childhood neglect was associated with 24-month retention in care; no other relationships between ACEs and HIV care outcomes were significant.

More research on the violence, mental health, and HIV triad, especially longitudinal studies with larger sample sizes among CWLH, is needed. Given the high co-occurrence of violence with some poor mental health and HIV outcomes across our sample, attempts to address one component of the triad without considering the other two may not be as effective as more comprehensive approaches. Interventions to improve HIV clinical outcomes would benefit from using a trauma-informed approach while aiming to improve mental health and refer CWLH to support services as needed. The first step toward such an approach is screening patients/clients for violence and related mental health concerns (e.g., PTSD). Specifically, given that so many forms of violence were related to PTSD (i.e., high co-occurrence with IPV, NPV, hate crimes, and ACEs), healthcare professionals working with CWLH could consider screening for PTSD. Rather than comprehensive violence screening, a short, efficient, low-burden PTSD screener may be a more effective, trauma-informed approach and reduce risk of re-traumatization. Upon identifying these needs, healthcare professionals may connect CWLH to the most appropriate violence and psychosocial support resources.

### 4.3 Violence screening

A trauma-informed approach to violence screening for CWLH involves several factors that must prioritize the woman’s individual needs and preferences. Depending on each woman’s circumstances, this may include ensuring that the person performing the screening is compassionate, discreet, trained, and ideally shares similar age, race, and gender characteristics. Additionally, screenings should ideally take place face-to-face at multiple points throughout the year, including during intake, in a safe and comfortable environment. Those conducting the screenings should caution against re-traumatization and consider inquiring about various forms of violence, not limited to physical or sexual IPV. Lastly, HIV care settings should actively inform CWLH about available support services for violence when they express interest. Depending on the woman’s choice, this could take the form of a general pamphlet for future reference or a direct referral to services.

The findings of this study indicate violence is highly prevalent among CWLH. Many have underscored the need to address this issue by calling for more integrated violence screening in HIV care settings; however, they have primarily focused on physical or sexual interpersonal violence (24–28). Considering the high prevalence of all forms of violence across our sample, as well as high co-occurrence with mental health symptoms (i.e., PTSD, depression, anxiety), a universal trauma-informed approach, including brief PTSD screening, within the HIV care continuum could provide more opportunities to recognize violence and link CWLH to appropriate support services (e.g., mental health services or safe housing programs), which in turn may increase engagement in HIV care. Despite the recognized need for screening, little is known about screening needs and preferences among CWLH. This may be especially important to understand in medically under-resourced populations (e.g., those seeking services at RWCs, ASOs, and safety-net hospitals), as these individuals are at increased risk of violence due to social and structural determinants, including poverty (66). To our knowledge, our study is the first to provide key insights into screening preferences among CWLH who receive care in these settings in the US South, the center of the HIV epidemic (1).

### 4.4 Strengths and limitations

Our study had several key strengths. Study team members involved in data collection completed comprehensive trauma-informed training with emphases on building rapport, maintaining confidentiality, and sharing violence support resources with participants as needed. These practices supported participant safety and greater data validity. Also, we recruited from various HIV care settings (i.e., clinics, ASOs, hospitals). We used mixed methods, including validated instruments, to examine multiple forms of violence and other HIV syndemic factors (i.e., mental health, substance use) (4, 68), capturing a diverse range of experiences CWLH may have. Our study is not without limitations. First, our sample only included two CWLH out-of-care (1.15%) despite recruiting from ASO and hospital-based settings. Compared to those engaged in care, out-of-care PLWH may be less likely to achieve viral suppression (69) and more likely to have depression or other mental health conditions (70). Hence, our study may not fully capture viral suppression and mental health among CWLH with experiences of violence, and the associations found between various forms of violence, mental health, and HIV outcomes may not hold among predominantly out-of-care CWLH populations. Another limitation was the reliance on self-reported data, which could introduce recall bias (e.g., misremembering exactly when violence occurred, such as past year versus lifetime IPV) and social desirability bias (e.g., underreporting substance use). Finally, our sample size was small, limiting our ability to perform multivariate analyses. As such, quantitative results should be interpreted with caution.

#### 4.4.1 Other factors related to HIV outcomes

Emerging evidence suggests resilience may impact relationships between violence/trauma, depression, and PTSD among CWLH, which in turn may impact HIV care outcomes (22, 52, 71). While we were unable to perform multivariate analyses to examine these potential relationships, our sample had a lower median resilience score (72) than the general US population (82, range 0–100) (73). However, this is somewhat consistent with other studies of CWLH; for example, a study of Canadian CWLH found a median resilience score of 64 (72), and another found a mean resilience score of 28.82 among US CWLH (71). Compared to others (presumably without HIV) who have experienced trauma, CWLH in our sample had higher resilience. For example, a 2012 study that also used the 25-item CD-RISC found mean scores of 61.3 and 74.3 among female abuse survivors with and without PTSD symptoms, respectively (74). It is unclear why CWLH in our sample had higher resilience, but it may explain the high proportions of viral suppression, as suggested by emerging research (22). Further research in this area is needed to better understand potential causal pathways. Additionally, according to SAVA syndemic theory, substance use may co-occur and interact with IPV and HIV outcomes (4). Though not highly prevalent, our sample included CWLH with high-risk or likely dependent alcohol consumption (10.23%) and past-year substance use, including cannabis (27.27%) and other drugs (18.18%, e.g., cocaine, heroin, narcotics). Considering violence, mental health, and HIV outcomes in a resilience and substance use context, our findings may not fully capture relationships between these three facets of health.

### 4.5 Implications and future directions

Multiple forms of violence were highly prevalent among CWLH, indicating a significant need for trauma-informed HIV care and violence screening tailored to individuals’ needs and preferences. Considering both our and other scholars’ findings regarding associations between violence, mental health, and HIV outcomes, recognizing and addressing violence as an upstream determinant of the HIV care continuum is critical. To successfully implement trauma-informed care and violence screening in HIV care settings, including RWCs, ASOs, and safety-net hospitals, it is necessary to consider the needs and preferences of both CWLH and healthcare professionals. Research in this area is promising (75), but gaps remain. Our study begins filling these gaps by identifying patient preferences. Aligning with these preferences, HIV care settings should consider regularly screening CWLH for multiple forms of violence in a trauma-informed manner, including creating an emotionally and physically safe space, having compassionate healthcare professionals conduct the screening, starting with questionnaires then following up during face-to-face discussions, and offering referrals to support services as needed. To continue understanding how to improve trauma-informed support services along the HIV care continuum, our team will next examine the acceptability and preferences of violence screening and support services implementation among healthcare professionals working in RWCs and community-based organizations.

## Data Availability

The raw data supporting the conclusions of this article will be made available by the authors, without undue reservation.
