# Supplementary material for: Violence experience among cis-gender women living with HIV in Atlanta, Georgia: impact on HIV-related health and their preferences for violence screening and support
Source: Front Public Health. 2025 Apr 14;13:1521493. doi: 10.3389/fpubh.2025.1521493 (PMC12034539; doi:10.3389/fpubh.2025.1521493)
Supplement: Supplementary file 1 [file Data_Sheet_1.docx]

**Supplemental Material: Measures for non-partner violence (NPV)**

*Crime-related NPV* was captured using four items, including having someone 1) “take something directed from you by using force or threat of force,”2) “attempt to rob you or actually rob you,” or 3/4) “attempt to or access in breaking into your home when you were (not there/there).”

*General NPV* was measured using 13 items, including ever having 1) “a serious accident at work, in a car, or somewhere else;” 2) “experienced a natural disaster such as a tornado, hurricane, flood or major earthquake, etc. where you felt you or your loved ones were in danger of death or injury;” 3) “experienced a man-made disaster such as a train crash, building collapse, bank robbery, fire, etc. where you felt you or your loved ones were in danger of death or injury;” 4) “been exposed to dangerous chemicals or radioactivity that might threaten your health;” 5) “been in any other situation in which you were seriously injured;” 6) “been in any other situation in which you feared you might be killed or seriously injured;” 7) “ever seen someone seriously injured or killed;” 8) “seen dead bodies (other than at a funeral) or had to handle dead bodies for any reason;” 9) “had a close friend or family member murdered, or killed by a drunk driver;” 10) “had a spouse, romantic partner, or child die;” 11) “had a serious or life-threatening illness;” 12) “received news of a serious injury, life-threatening illness, or unexpected death of someone close to you;” and 13) “had to engage in combat while in military service in an official or unofficial war zone.”

*Physical/sexual NPV* was measured using seven items, including 1) being made to “have intercourse or oral or anal sex against your will;” 2) having someone who “touched private parts of your body, or made you touch theirs, under force or threat;” 3) “situations in which another person tried to force you to have unwanted sexual contact” other than those that were captured by the previous two items; 4) ever being attacked by someone (including family or friends) with a gun, knife, or other weapon; 5) ever being attacked by someone (including family or friends) without a weapon; 6) ever being beaten, spanked, or pushed by a family member hard enough to cause injury; and 7) any other extraordinarily stressful situation or event that was not covered in a previous question.
